# The Wear Resistance of NiCrSiB-20%CaF_2_ Sinters in the Temperature Range 23–600 °C

**DOI:** 10.3390/ma18071405

**Published:** 2025-03-21

**Authors:** Adam Piasecki, Mateusz Kotkowiak, Oleksandr Tisov, Bartosz Gapiński, Michał Jakubowicz, Julia Sobkowiak, Maciej Tuliński, Stanisław Legutko

**Affiliations:** 1 Faculty of Materials Engineering and Technical Physics, Institute of Materials Science and Engineering, Poznan University of Technology, Jana Pawla II 24, 61-139 Poznan, Poland; mateusz.kotkowiak@put.poznan.pl (M.K.); juliasobkowiak5@gmail.com (J.S.); maciej.tulinski@put.poznan.pl (M.T.); 2 School of Aerospace Engineering, Xi’an Jiaotong University, West Xianning Road 28, Xi’an 710049, China; oleksandrtisov@xjtu.edu.cn; 3 Faculty of Mechanical Engineering, Institute of Mechanical Technology, Poznan University of Technology, Piotrowo 3, 61-138 Poznan, Poland; bartosz.gapinski@put.poznan.pl (B.G.); michal.jakubowicz@put.poznan.pl (M.J.); stanislaw.legutko@put.poznan.pl (S.L.)

**Keywords:** self-lubricant, calcium fluoride, nickel alloy, tribofilm, wear mechanism

## Abstract

In this work, powder metallurgy was used in order to produce self-lubricating composite materials. The NiCrSiB alloy as a matrix of the sinters and 20 wt. % CaF_2_ as a solid lubricant were used. The sinters were subjected to wear tests using the pin-on-disc method at four different temperatures (room temperature (RT), 200, 400, and 600 °C). The coefficients of friction of the friction pairs were determined, and research on their wear mechanism was carried out. For this purpose, research techniques such as Light Microscopy (LM), Scanning Electron Microscopy (SEM), Energy Dispersive Spectroscopy (EDS), X-ray photoelectron spectroscopy (XPS), X-ray Diffraction (XRD), and profilometer were used. Based on the conducted tests, it was found that CaF_2_ was smeared on the surfaces of the samples and counter-specimens, particularly at elevated temperatures. Moreover, it was found that micro-cutting and micro-ploughing are the major wear of the friction pairs at room temperature, while with the increasing temperature, they were dominated by the reduction of such mechanisms, which is associated with the formation of a tribofilm composed of CaF_2_ and oxidation wear.

## 1. Introduction

Friction wear is one of the main causes of damage to machine parts and tools. It is estimated that its occurrence is as high as 80%. The reduction of friction wear by improving the sliding properties can be achieved using lubricants. Lubricating oils contaminate the environment during their production and in practically all stages of their application: transport to end-users, long-term storage, actual application, and utilization. The primary components that are hazardous to people are oils, aromatic and unsaturated hydrocarbons, and heterogenous compounds (containing sulphur, nitrogen, and oxygen). In recent years, self-lubricating materials and coatings have been rapidly developing [1]. Lubricants contain some of the solid components described and divided into three groups, depending on the work temperature [2]: from −200 °C to room temperature, from room temperature to 500 °C, and above 500 °C. Conventional lubricants including soft metals, transition metal dichalcogenides (e.g., molybdenum and tungsten disulphides [3], diselenides, and ditellurides), graphite [4,5], and polymers (i.e., polytetrafluoroethylene, polyimides) [6] lubricate effectively at temperatures below 500 °C. Typical examples of high-temperature lubricants (above 500 °C) are oxides (e.g., ZnO, PbO, CuO, B_2_O_3_, ReO_2_) [7], fluorides (CaF_2_, BaF_2_, CeF_2_, LaF_3_) [8,9,10,11], sulphates (e.g., CaSO_4_, BaSO_4_, SrSO_4_) and the combination of some of them [2,12,13]. Various methods are applied to produce self-lubricating materials and coatings. Self-lubricating materials are usually sintered with the use of cold compaction and heating [14,15,16,17,18], cold compaction and hot pressing [19,20,21], hot pressing only [22,23,24,25], microwave and pulse electric sintering [26], or spark-plasma sintering (SPS) [27,28,29,30]. The tribological properties of sintered high-speed steels could also be improved by the addition of self-lubricants [31]. In ceramic composites on metal-base alloys, graphite [32,33,34,35,36,37,38], lead [39], silver [40,41,42], and MoS_2_ [43] are often used as solid lubricants. Self-lubricating coatings are mainly formed by the following methods: high velocity oxygen fuel (HVOF) spraying [44,45], plasma spraying [46,47,48,49], thermal spraying [50], low-pressure cold spraying [51], laser cladding [52,53,54,55,56], PLD [2], and laser alloying [57,58,59]. Apart from the self-lubricating layers and coatings, self-lubricating materials are produced using powder metallurgy. This technique guarantees the obtaining of uniform materials with a regular distribution of each component in the microstructure. Nickel alloys gained their popularity mainly due to oxidation resistance at high temperatures. However, due to their low wear resistance to friction, their use is limited. Currently, many research centers are conducting research on the surface modification of nickel alloys or nickel-based sinters in order to improve their tribological properties [60,61]. In the present work, the NiCrSiB alloy was modified by adding a solid lubricant in the form of CaF_2_. The wear mechanism from room temperature to 600 °C was investigated. In the available literature, no information was found on the testing of these alloys containing additives of solid lubricants. There is therefore insufficient knowledge about the mechanism of wear and friction of these alloys. NiCrSiB powder is used as an additional material during the hardfacing and thermal spraying processes [62,63,64]. The coatings are characterized by high wear resistance to friction and are often used on agricultural tools working in the soil. In this case, there is no problem of lubrication. In addition, padded coatings are characterized by a certain share of the substrate, while sprayed coatings are characterized by porosity and an adhesive bond between the coating and the substrate.

In this study, friction pairs were used where the nickel alloy is also the counter-specimen. The aim of the research was to determine the wear mechanisms of NiCrSiB nickel alloy-based sinters with and without CaF_2_ addition in the range from room temperature to 600 °C. The problems of lubrication and reduction of the coefficient of friction are important in this case, and the research on the wear mechanism may contribute to better use of these materials in the industry sector, particularly in such work at elevated and high temperatures, under dry friction conditions.

## 2. Materials and Methods

### 2.1. Sinter Preparation

**Figure 1:**
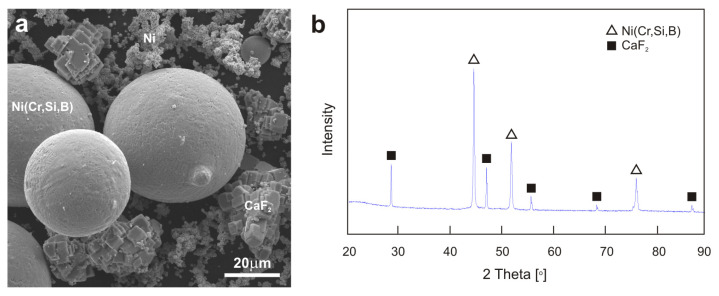
Morphology (**a**) and XRD pattern (**b**) of Ni-NiCrSiB-CaF_2_ powder mixture.

In the first stage, the powder mixtures were prepared in the drum for 1 h. Figure 1 presents the morphology and the XRD pattern of the powder mixture. The pure nickel powders (99.7% purity, Sigma Aldrich, Saint Louis, MO, USA) were characterized by a dendritic structure, and the powder grains of the NiCrSiB alloy had a sphere-like shape. The CaF_2_ solid lubricant powders (Sigma Aldrich, Saint Louis, MO, USA) had a cubic structure. The Ni95.5Cr0.5Si2.5B1.5 powder (Euromat Sp. z o.o., Wrocław, Poland) is a solid solution of chromium, silicon, and boron in nickel. The XRD analysis did not indicate the presence of other phases. The peak from pure nickel overlaps with the peak from the solid Ni (Cr, Si, B) solution. In the second stage, the moldings of the powders were produced (50% pure nickel and 50% NiCrSiB alloy as well as 40 wt. % pure nickel, 40 wt. % NiCrSiB alloy, and 20 wt. % CaF_2_). Based on previous tests performed on self-lubricating surface layers and sinters, the CaF_2_ content in the sintered material was set at 20 wt. % [8,9,10,18,57,58,59]. Cold pressing of the powder mixtures was performed under the pressure of 1.17 GPa using an automatic hydraulic press (MP250, Maassen GmbH, Möglingen, Germany). In the next stage, the sintering process was carried out of the moldings at a temperature of 1100 °C for 3 h in a tube furnace under argon atmosphere (RT 50/250, Nabertherm GmbH, Lilienthal, Germany). The cooling of the sinters was carried out in a furnace at a rate of 250 °C/h. The produced sinters were 4 mm in diameter and 5 mm in height.

### 2.2. Microstructure and Hardness

The microstructures of the sinters were observed through the light microscope (LAB 40, OPTA-TECH, Warsaw, Poland). A diffractometer (Empyrean, PANalytical, Almelo, The Netherlands) equipped with a copper lamp (X-ray wavelength λ = 1.54060 Å) and an X’Celerator detector were used to test the phase composition of the powders and sinters. The diffractograms were recorded at room temperature in an angular range of 20–90° 2Theta in steps of 0.0170°. The phase analysis of the obtained diffractograms was carried out in the HighScore Plus software (version 5.3) equipped with the PDF-5+ database (2024 version). The hardness was measured with a Vickers hardness tester (Falcon 500, INNOVATEST, Maastricht, The Netherlands) at a static load of 2 kgf, with a dwell time of 15 s. Eight hardness measurements were made. The metallographic observations and the hardness tests were performed on metallographic specimens prepared from the sinters. The metallographic specimens were ground with the abrasive paper of a decreasing grain size and were finally polished with Al_2_O_3_. The porosity was determined on SEM images of microstructures using computer software, which is an integral part of the microscope software. Ten areas on the metallographic sections were analyzed during direct observation on a scanning electron microscope (Mira 3, Tescan, Brno, Czech Republic), and the result was calculated as the ratio of the pore areas against the entire area of the images.

### 2.3. Wear Tests

The pin-on-disk high-temperature tribometer (T-21, Lukasiewicz Research Network—IST, Radom, Poland) was used to measure the tribology properties of the sintered materials. The temperature of the tests was 23, 200, 400, and 600 °C. The counter-specimens were made of commercially available Inconel^®^625 nickel alloy (purchased from BIBUS METALS Sp. z o.o., Dąbrowa, Poland). They had a disc shape, 25.4 mm in diameter and 4 mm in thickness. The friction tests under dry friction conditions were carried out at a load of 5 N for 1 h at a rotational speed of 120 min^−1^. Each of the wear tests was repeated three times. During the tests, the friction forces were recorded for the following friction pair: sintered material (pin)—Inconel^®^625 (disc). From that data, the coefficients of friction were calculated using the formula below:

$$\mu =\frac{F_{f}}{F_{l}}$$

where *μ* is coefficient of friction, *F_f_* is friction force [N], and *F_l_* is load applied [N].

The results of the wear tests have been shown as graphs of the coefficients of friction as a function of the test times.

### 2.4. Worn Surface Studies

The worn surface of the sintered materials and the counter-specimens after the wear tests were observed using SEM (Mira 3, Tescan, Brno, Czech Republic) equipped with EDS (Ultim Max 65, Oxford Instruments, High Wycombe, UK). An accelerating voltage of 12 kV was applied. The contents of the elements such as Ni, Cr, Si, Ca, F, and O were analyzed. Due to the low atomic number and the small amount of boron in the NiCrSiB powder, its content was not measured. The distribution of the element concentrations in the form of EDS traditional pattern maps and color scale were performed too. In the color scale, the colors correspond to the concentration of a selected element (white pixels denote the highest concentration of an element, and the black areas denote zero concentration). The XPS tests were performed under high vacuum conditions (multi-chamber UHV system, Prevac Sp. z o.o., Rogów, Poland). The source of excitation radiation was an aluminum (Al) anode generating monochromatic radiation with a characteristic Al Kα line and the energy of 1486.7 eV. Moreover, topography measurements of the friction track on the counter-specimens were performed by the profilometer (Hommel T8000, Jenoptik, Jena, Germany) with a diamond tip with a radius of 2 µm.

## 3. Results

### 3.1. Microstructure and Hardness of the Sinters

**Figure 2:**
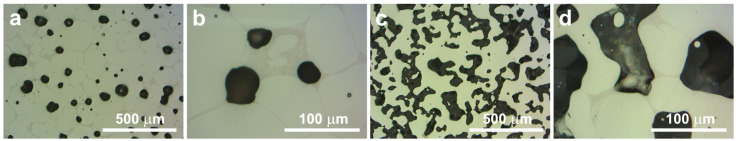
Microstructure of the NiCrSiB (**a**,**b**) and NiCrSiB + 20%CaF_2_ (**c**,**d**) sinters.

**Figure 3:**
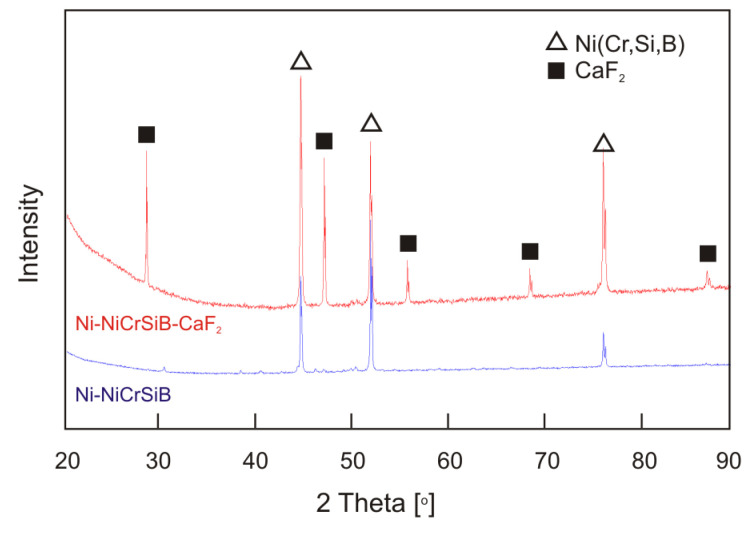
XRD pattern of sinters.

The microstructures of the produced sinters are shown in Figure 2. In both cases, the matrix microstructure is the NiCrSiB alloy with the addition of pure nickel. For both sinters, the microstructure includes light spherical grains and the presence of eutectic between them, probably composed of nickel and a phase with a chemical composition similar to that of the NiCrSiB alloy, originating from the melted NiCrSiB alloy grains. The resulting eutectic binds individual grains together. The introduction of pure nickel was intended to achieve a greater degree of sintering of the NiCrSiB alloy grains. The purpose of introducing the NiCrSiB alloy was to achieve a higher hardness of the sinter compared to pure nickel sinter-66 HV0.05 [18]. In the case of a sintered material containing only nickel and the NiCrSiB alloy, characteristic spherical pores of a size of up to approximately 120 µm are visible in the sinter microstructure, and the porosity is 11%. The introduction of a solid lubricant in the form of calcium fluoride into the sinter significantly reduced the porosity, which was approximately 2%. In the spaces formed between the sintered grains of nickel and the NiCrSiB alloy powders, CaF_2_ particles can be observed, evenly distributed in the matrix. Visible porosity in the spots where solid lubricant is present is related to its washing out during the preparation of the metallographic specimen. Thus, calcium fluoride fills almost all the spaces between the grains of the sintered nickel and the NiCrSiB alloy powders. The metal matrix constitutes a skeleton. The skeleton is correct, free of cracks, and the porosity is negligible. The NiCrSiB + CaF_2_ sinter is characterized by a lower hardness of 79.8 HV2 compared to the sinter without the addition of calcium fluoride (91.5 HV2). Compared to the pure nickel sinter, an increase in the hardness of approximately 39% was achieved for the NiCrSiB sinter and approximately 21% for the NiCrSiB sinter with a 20% addition of calcium fluoride. The phase composition studies of the sinters have not revealed the formation of new phases other than the components of the powder mixtures during the sintering process (Figure 3).

### 3.2. Wear Resistance

**Figure 4:**
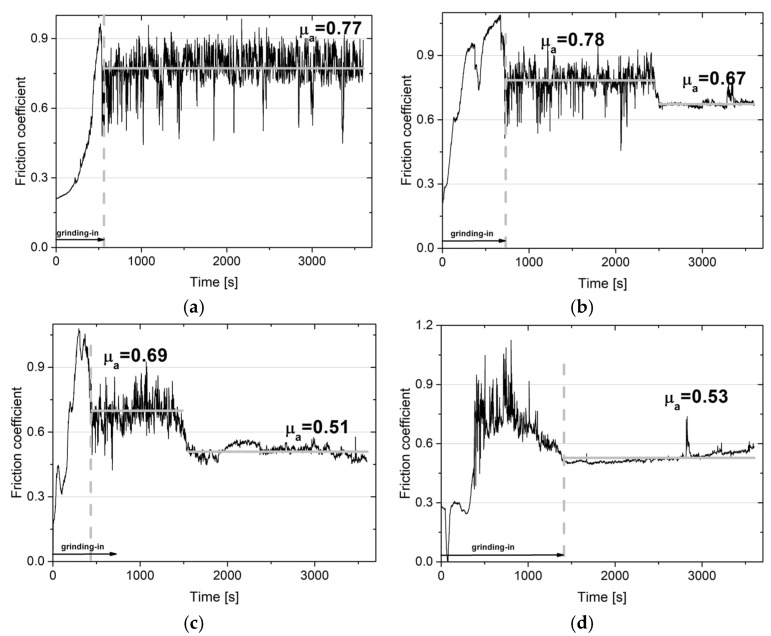
Coefficient of friction vs. time of friction for the NiCrSiB–Inconel^®^625 alloy friction pairs at RT (**a**), 200 °C (**b**), 400 °C (**c**), and 600 °C (**d**).

**Figure 5:**
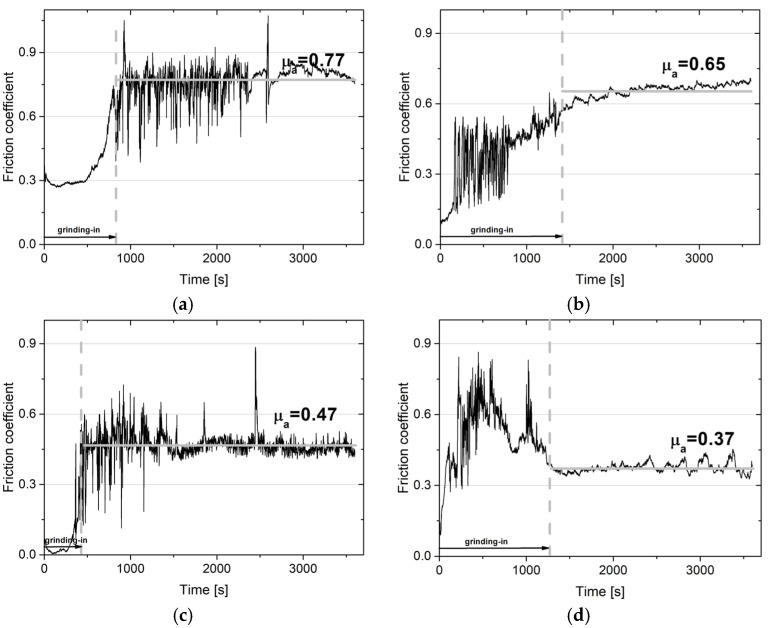
Coefficient of friction vs. time of friction for the NiCrSiB + 20% CaF_2_–Inconel^®^625 alloy friction pairs at RT (**a**), 200 °C (**b**), 400 °C (**c**), and 600 °C (**d**).

**Figure 6:**
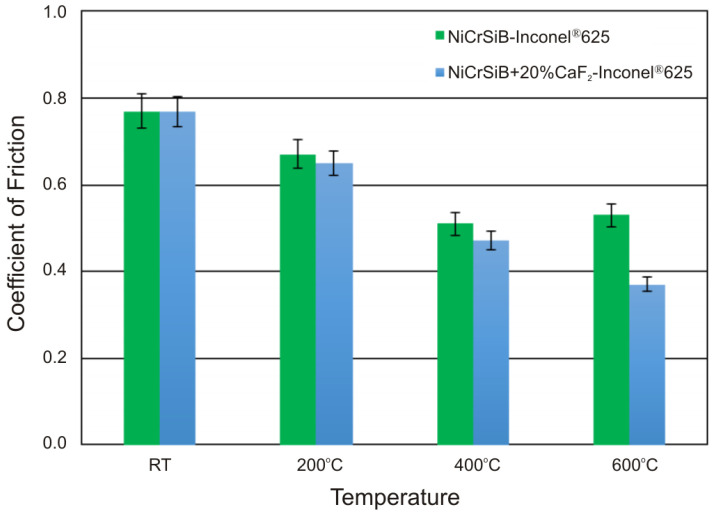
Coefficients of friction.

The wear resistance tests were performed at room temperature, 200, 400, and 600 °C. Figure 4 and Figure 5 show the change in the coefficient of friction as a function of the test time, which was 3600 s. The average values of the friction coefficients determined over the stabilized period following three tests are shown in Figure 6. The change in the coefficient of friction at room temperature for the NiCrSiB sinter is characterized by quite significant fluctuations throughout the test range. The average coefficient of friction was 0.77 (Figure 4a). In the case of the sinter containing calcium fluoride (Figure 5a), the average coefficient of friction has a similar value, with small fluctuations in the COF value observed after approx. 2500 s, which may indicate a positive effect of CaF_2_ and its partial smearing. A similar behavior of the coefficients of friction was observed in [18], where the coefficients of friction of nickel-based sinters were also analyzed in the temperature range from room temperature to 600 °C. At the test temperature of 200 °C (Figure 4b and Figure 5b), similar coefficients of friction were obtained for the friction pairs mating with sinters with and without the CaF_2_ addition, and after the grinding-in period they were 0.67 and 0.65, respectively. However, it is noteworthy that for the NiCrSiB sinter, a period of large COF fluctuations and an average coefficient of friction of 0.78 was observed (Figure 4b). The coefficients of friction of the friction pairs tested at a temperature of 400 °C were 0.51 and 0.47, respectively, for the friction pair, in which the sintered material was used with and without a solid lubricant. At the test temperature of 600 °C, the coefficient of friction for the NiCrSiB + 20% CaF_2_–Inconel^®^625 friction pair was the lowest and equaled 0.37. For the friction pair without the addition of CaF_2_ in the sinter, a coefficient of friction of 0.53 was recorded. With reference to [18], the coefficients of friction for all NiCrSiB + 20% CaF_2_–Inconel^®^625 friction pairs were lower compared to the Ni + 20% CaF_2_–Inconel^®^625 ones.

**Figure 7:**
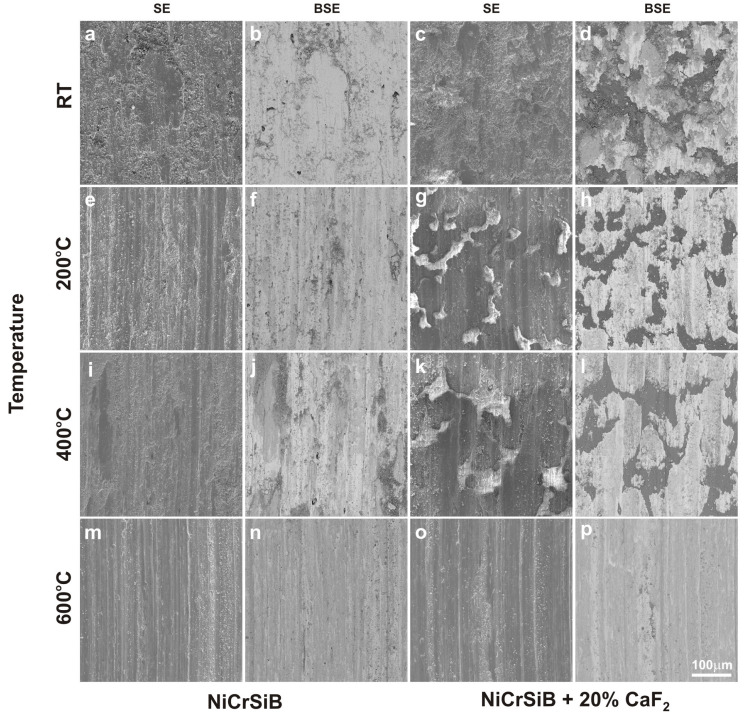
Worn surface of the sinters after friction wear test at room temperature (**a**–**d**), 200 °C (**e**–**h**), 400 °C (**i**–**l**), and 600 °C (**m**–**p**).

**Figure 8:**
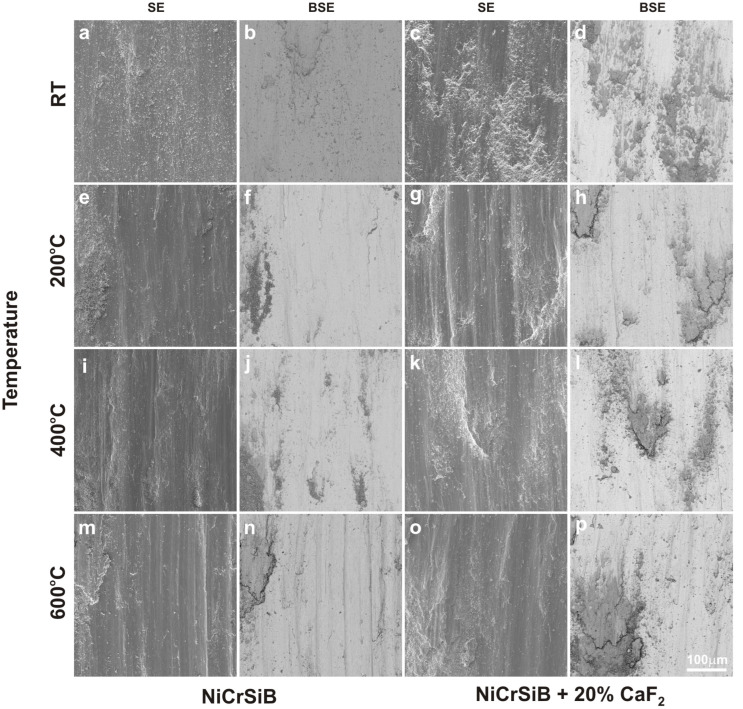
Worn surface of the counter-specimens after friction wear test at room temperature (**a**–**d**), 200 °C (**e**–**h**), 400 °C (**i**–**l**), and 600 °C (**m**–**p**).

**Figure 9:**
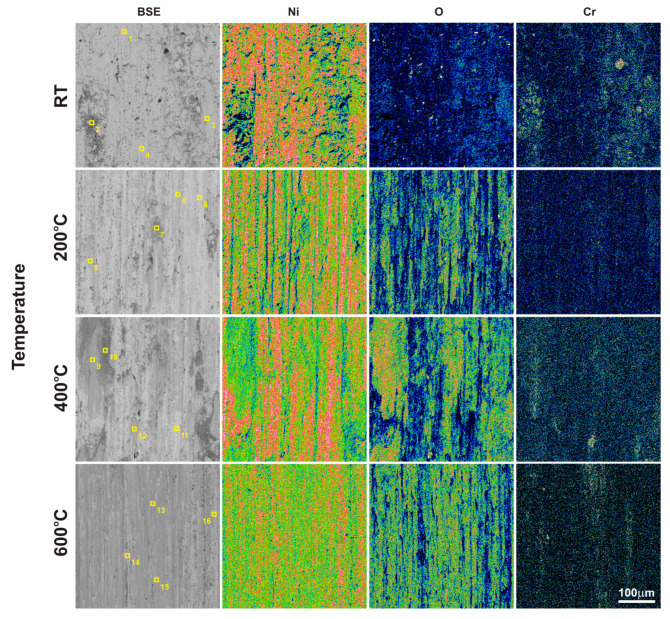
SEM images with marked areas of EDS analysis (according to Table 1) and EDS maps of the element concentration distributions on the NiCrSiB sinter surface after the friction wear test.

**Figure 10:**
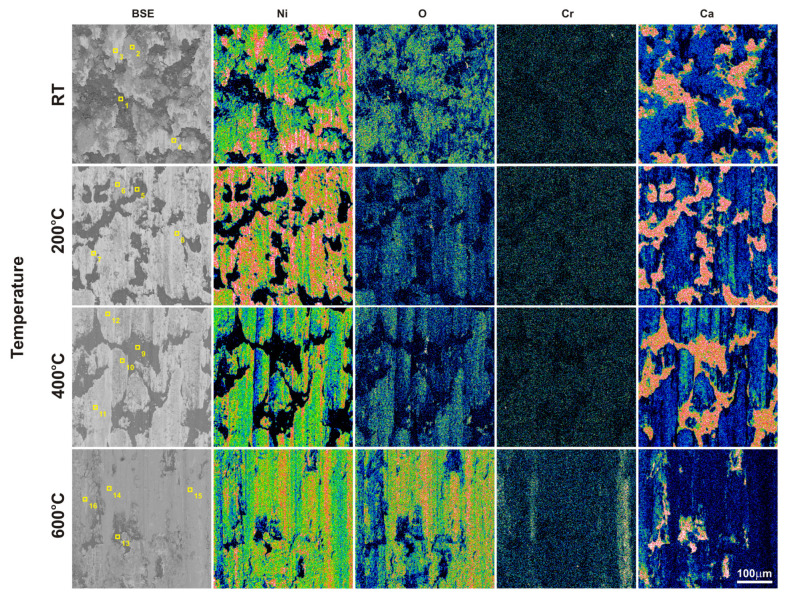
SEM images with marked areas of EDS analysis (according to Table 2) and EDS maps of the element concentration distributions on the NiCrSiB + 20%CaF2 sinter surface after the friction wear test.

**Figure 11:**
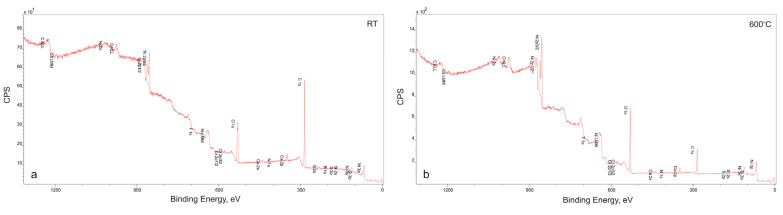
XPS patterns of the NiCrSiB-20 wt. %CaF_2_ sinter surface after the friction wear test, tested at RT (**a**) and 600 °C (**b**).

**Figure 12:**
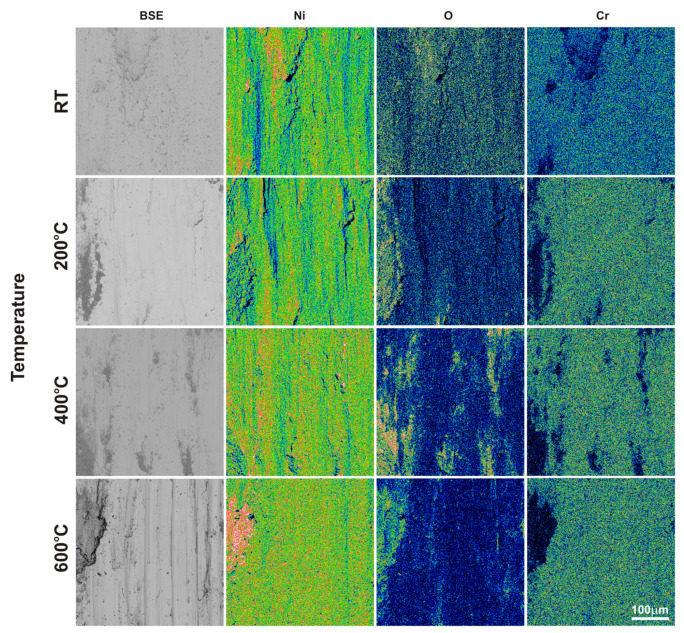
EDS maps of the element concentration distributions on the Inconel^®^625 surface after the friction wear test with the NiCrSiB sinter.

**Figure 13:**
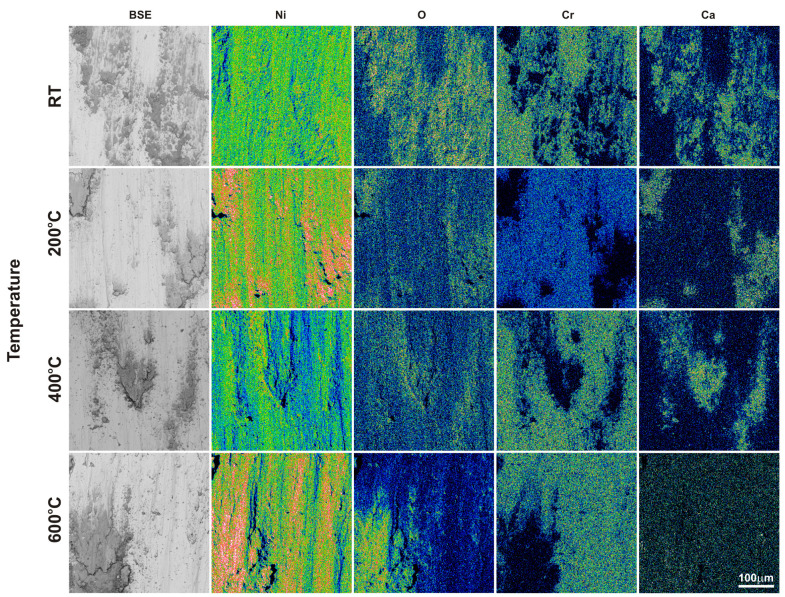
EDS maps of the element concentration distributions on the Inconel^®^625 surface after the friction wear test with the NiCrSiB + 20%CaF_2_ sinter.

**Figure 14:**
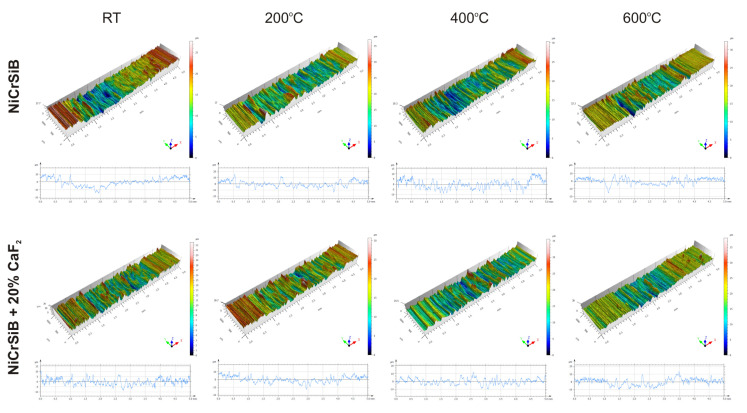
Surface topography measurements of the counter-specimens.

After the friction wear tests, SEM observations of the sinters and counter-specimen surfaces were performed (Figure 7 and Figure 8). These observations were supplemented by EDS X-ray microanalysis studies, X-ray photoelectron spectroscopy, and surface topography (Figure 9, Figure 10, Figure 11, Figure 12, Figure 13 and Figure 14, Table 1 and Table 2). The SEM images were taken in secondary electron (SE) and backscattered electron (BSE) contrast. In the BSE images, the darker areas contain light elements, and the brighter areas contain heavier elements. For the tests carried out at room temperature, traces of micro-cutting and micro-ploughing were observed on the friction surfaces of all sinters (Figure 7a–d). As the test temperature increases, the surfaces become smoother, and traces of micro-cutting and micro-ploughing can also be observed. The chemical composition tests showed the presence of oxygen on the friction surfaces of the sinters, which indicates the occurrence of oxidation wear (Figure 9 and Figure 10). Its content is higher at higher temperatures (Table 1 and Table 2).

**Table 1:** The chemical composition of the worn surfaces of the NiCrSiB sinters (according to Figure 9).

Temperature	Point	Ni	Cr	Si	Ca	F	O
23 °C	1	95.6	1.5	1.4	-	-	1.4
	2	90.4	7.4	0.6	-	-	1.6
	3	85.8	8.0	1.2	-	-	5.1
	4	96.7	1.0	1.4	-	-	0.9
200 °C	5	84.2	2.2	1.3	-	-	12.3
	6	94.3	1.0	1.5	-	-	3.2
	7	87.4	3.1	1.0	-	-	8.6
	8	84.7	2.2	1.3	-	-	11.8
400 °C	9	73.8	5.1	1.1	-	-	20.0
	10	82.0	2.8	1.1	-	-	14.1
	11	96.7	0.3	1.6	-	-	1.4
	12	80.5	1.8	1.3	-	-	16.3
600 °C	13	80.0	2.9	0.8	-	-	16.2
	14	95.6	0.2	1.4	-	-	2.7
	15	82.9	1.5	0.9	-	-	14.8
	16	93.0	0.3	1.4	-	-	5.3

**Table 2:** The chemical composition of worn surfaces of the NiCrSiB + 20%CaF2 sinters (according to Figure 10).

Temperature	Point	Ni	Cr	Si	Ca	F	O
23 °C	1	0.5	0.0	0.0	46.9	52.0	0.5
	2	70.2	0.2	1.4	10.7	8.9	8.6
	3	94.5	0.1	2.1	0.9	1.3	1.1
	4	81.6	0.1	1.7	5.8	5.8	5.1
200 °C	5	1.1	0.0	0.0	44.0	54.2	0.6
	6	71.5	0.2	1.2	11.5	11.1	4.5
	7	90.5	0.2	1.7	3.1	3.6	1.0
	8	77.1	0.2	1.4	7.9	8.7	4.7
400 °C	9	0.4	0.0	0.0	43.8	55.2	0.6
	10	63.2	0.1	1.5	14.0	16.7	4.5
	11	89.4	0.2	1.8	3.3	3.4	1.9
	12	73.5	0.2	1.5	9.8	10.1	5.0
600 °C	13	6.7	0.1	0.1	45.0	46.0	2.3
	14	70.9	4.0	0.9	3.1	3.7	17.5
	15	72.6	4.5	1.0	2.6	2.9	16.4
	16	80.1	1.0	1.4	3.5	4.3	9.7

In the case of the sinters containing calcium fluoride, its smearing is visible even at room temperature (Figure 7d and Figure 10). For these sinters, the decomposition of the CaF_2_ additive and its smearing on the sinter surface are very clearly visible after the tests at temperatures of 200 and 400 °C (Figure 7g,h,k,l). These observations were confirmed by the chemical composition studies in the form of EDS maps of the distribution of element concentrations and point analysis. The surface of the NiCrSiB + 20%CaF_2_ sinter after the friction wear test at 600 °C in the BSE contrast (Figure 7p) is characterized by a uniform grey color, which may indicate that a tribofilm of similar thickness was obtained on its surface. The fluorine and calcium contents outside the spots where only CaF_2_ is added are in the range of 2.6–3.5 wt. % and 2.9–4.3 wt. %, respectively (Table 2). Due to the limitations of the EDS method, the boron content was not determined. Figure 11 shows the results of the XPS analysis performed on the surfaces of the sintered materials containing calcium fluoride after the friction wear tests at room temperature (Figure 11a) and 600 °C (Figure 11b). The peaks of Ca, F, Ni, and O are visible following both wear tests. The analysis proved that a tribofilm layer had formed on the surface of the samples. Also, it was confirmed that oxidative wear occurred, having a major impact, particularly at an elevated temperature. Based on the observations of the surfaces of the counter-specimens, it was found that the main mechanism of their wear is micro-cutting and micro-ploughing (Figure 8). Based on the examination of the chemical composition of the wear surface of the counter-specimens in the form of EDS maps of the distribution of element concentrations (Figure 12 and Figure 13), it was found that wear through oxidation is also a wear mechanism, both for the samples with and without the addition of CaF_2_. The uniform distribution of calcium on the surface of the counter-specimens after testing at 600 °C indicates the smearing of the CaF_2_ additive and the formation of a tribofilm of a similar thickness. At the remaining test temperature, smearing of calcium fluoride on the surfaces of the counter-specimens was also observed, but its presence was not uniform. The surface topography measurements have been shown in Figure 14. These tests confirmed that the main wear mechanism of the counter-specimens is abrasive wear. The individual profiles have a concave shape with greater wear observed for the counter-specimens mating with the sinters without calcium fluoride. A slight abrasion of the counter-specimens mating with the sinters containing CaF_2_ proves its positive effect. Such profiles prove very good tribological properties of the friction pairs. Visible abrasion probably occurs at the initial stage of the testing, i.e., during grinding-in. After this stage, calcium fluoride is released, which has a beneficial effect on the next stage of the friction and reduces the wear of the friction pairs. The tests performed at a higher temperature do not increase the abrasion of the counter-specimens, which is related to the smearing of calcium fluoride between the mating surfaces and the formation of a tribofilm of a similar thickness at the test temperature of 600 °C.

## 4. Conclusions

In this work, NiCrSiB and NiCrSiB with 20 wt. % CaF_2_ sinters produced by powder metallurgy were examined. The following conclusions have been drawn from the study:

- The sinter with the calcium fluoride is characterized by a lower porosity compared to the sinter without this additive and is approximately 2% and 11%, respectively.
- The sinters with evenly distributed calcium fluoride in the metal matrix were obtained.
- The sinter containing 20 wt. % CaF_2_ is characterized by a lower hardness of 79.78 HV2 compared to the sinter without the addition of calcium fluoride (91.48 HV2).
- The coefficients of friction were lower at each test temperature for the friction pairs mating with the sinters containing calcium fluoride additives and decreased with the increasing test temperature. The lowest coefficient of friction (0.37) was observed for the Inconel^®^625–NiCrSiB + 20% CaF_2_ friction pair at the test temperature of 600 °C.
- The main wear mechanisms of the Inconel^®^625–NiCrSiB friction pairs were micro-cutting, micro-ploughing and oxidation wear, with the wear intensification being lower at higher temperatures.
- The main wear mechanisms of the Inconel^®^625–NiCrSiB + 20%CaF_2_ friction pairs were micro-cutting, micro-ploughing, and oxidation wear. However, due to the presence of calcium fluoride, the intensification of these mechanisms was lower due to the tribofilm formation during the test, particularly at a temperature of 600 °C.

## Data Availability

The original contributions presented in this study are included in the article. Further inquiries can be directed to the corresponding author.

## References

[1] Kumar A., Kumar M., Tailor S. (2023). Self-Lubricating Composite Coatings: A Review of Deposition Techniques and Material Advancement. Mater. Today Proc..

[2] Yang J.-F., Jiang Y., Hardell J., Prakash B., Fang Q.-F. (2013). Influence of Service Temperature on Tribological Characteristics of Self-Lubricant Coatings: A Review. Front. Mater. Sci..

[3] Piasecki A., Kotkowiak M., Tulinski M., Čep R. (2022). Tribological Properties of Cu-MoS_2_-WS_2_-Ag-CNT Sintered Composite Materials. Materials.

[4] Çelik A., Özer H.Ö., Tüzemen Ş.M., Yıldız M., Kovacı H. (2023). Synthesis, Characterization and Tribological Properties of Solid Lubricant Graphite Films Produced by PECVD. Mater. Today Commun..

[5] Somberg J., Gonçalves G., Emami N. (2023). Graphene Oxide versus Graphite and Chemically Expanded Graphite as Solid Lubricant in Ultrahigh Molecular Weight Polyethylene Composites. Tribol. Int..

[6] Belyak O.A., Kolesnikov I.V., Suvorova T.V. (2023). Modeling of Tribological Properties of Self-Lubricating Composite Materials. Lecture Notes in Mechanical Engineering.

[7] Essa F.A., Zhang Q., Huang X., Ibrahim A.M.M., Ali M.K.A., Abdelkareem M.A.A., Elagouz A. (2017). Improved Friction and Wear of M50 Steel Composites Incorporated with ZnO as a Solid Lubricant with Different Concentrations under Different Loads. J. Mater. Eng. Perform..

[8] Kotkowiak M., Piasecki A. (2022). Characterization of Wear Properties of Pure Nickel Modified by Ni-Cr Composite and CaF_2_ Solid Lubricant Addition. Materials.

[9] Kotkowiak M., Piasecki A., Kotkowiak M., Buchwald T. (2022). The Mechanism of Wear Reduction in the Ni-CaF_2_ Composite Material: Raman and Confocal Microscopy Insights. Materials.

[10] Piasecki A., Kotkowiak M., Kulka M. (2017). The Effect of CaF_2_ and BaF_2_ Solid Lubricants on Wear Resistance of Laser borided 100CrMnSi6-4 Bearing Steel. Arch. Mater. Sci. Eng..

[11] Roszak M.R., Kurzawa A., Roik T., Gavrysh O., Vitsiuk I., Barsan N., Pyka D., Bocian M., Jamroziak K. (2023). Friction Films Analysis and Tribological Properties of Composite Antifriction Self-Lubricating Material Based on Nickel Alloy. Mater. Sci. Pol..

[12] Essa F.A., Zhang Q., Huang X., Kamal M., Elagouz A., Abdelkareem M.A. (2017). Effects of ZnO and MoS_2_ Solid Lubricants on Mechanical and Tribological Properties of M50-Steel-Based Composites at High Temperatures: Experimental and Simulation Study. Tribol. Lett..

[13] Essa F.A., Zhang Q., Huang X. (2017). Investigation of the Effects of Mixtures of WS_2_ and ZnO Solid Lubricants on the Sliding Friction and Wear of M50 Steel against Silicon Nitride at Elevated Temperatures. Wear.

[14] Muthuraja A., Senthilvelan S. (2015). Development of Tungsten Carbide Based Self Lubricant Cutting Tool Material: Preliminary Investigation. Int. J. Refract. Met. Hard Mater..

[15] Yang X., Wang Z., Song P., Cheng J., Gu J., Ma T. (2014). Dry Sliding Wear Behavior of Al_2_O_3_-TiC Ceramic Composites Added with Solid Lubricant CaF_2_ by Cold Pressing and Sintering. Tribol. Trans..

[16] Ding C.-H., Liu C.-H., Yang Z.-M., Wang Y.-P., Sun Z.-B., Yu L. (2010). Effect of Size Refinement and Distribution of Lubricants on Friction Coefficient of High Temperature Self-Lubricating Composites. Compos. Sci. Technol..

[17] Konopka K., Roik T.A., Gavrish A.P., Vitsuk Y.Y., Mazan T. (2012). Effect of CaF_2_ Surface Layers on the Friction Behavior of Copper-Based Composite. Powder Metall. Met. Ceram..

[18] Kotkowiak M., Piasecki A., Kulka M. (2019). The Influence of Solid Lubricant on Tribological Properties of Sintered Ni–20% CaF_2_ Composite Material. Ceram. Int..

[19] Deng J., Li L., Yang X., Liu J., Sun J., Zhao J. (2007). Self-Lubrication of Al_2_O_3_/TiC/CaF_2_ Ceramic Composites in Sliding Wear Tests and in Machining Processes. Mater. Eng..

[20] Deng J., Cao T., Yang X., Liu J. (2006). Self-Lubrication of Sintered Ceramic Tools with CaF_2_ Additions in Dry Cutting. Int. J. Mach. Tools Manuf..

[21] Xu C.-Y., Wu G., Xiao G., Fang B. (2014). Al_2_O_3_/(W,Ti)C/CaF_2_ Multi-Component Graded Self-Lubricating Ceramic Cutting Tool Material. Int. J. Refract. Met. Hard Mater..

[22] Deng J., Cao T. (2007). Self-Lubricating Mechanisms via the in Situ Formed Tribofilm of Sintered Ceramics with CaF_2_ Additions When Sliding against Hardened Steel. Int. J. Refract. Met. Hard Mater..

[23] Kong L., Zhu S., Bi Q., Qiao Z., Yang J., Liu W. (2014). Friction and Wear Behavior of Self-Lubricating ZrO_2_(Y_2_O_3_)–CaF_2_–Mo–Graphite Composite from 20 °C to 1000 °C. Ceram. Int..

[24] Cui G., Lu L., Wu J., Liu Y., Gao G. (2014). Microstructure and Tribological Properties of Fe–Cr Matrix Self-Lubricating Composites against Si_3_N_4_ at High Temperature. J. Alloys Compd..

[25] Song P., Yang X., Wang S., Yang L. (2014). Tribological Properties of Self-Lubricating Laminated Ceramic Materials. J. Wuhan Univ. Technol.-Mater. Sci. Ed..

[26] Kim S.-H., Wohn Lee S. (2014). Wear and Friction Behavior of Self-Lubricating Alumina–Zirconia–Fluoride Composites Fabricated by the PECS Technique. Ceram. Int..

[27] Ouyang J.H., Sasaki S., Murakami T., Umeda K. (2004). The Synergistic Effects of CaF_2_ and Au Lubricants on Tribological Properties of Spark-Plasma-Sintered ZrO_2_(Y_2_O_3_) Matrix Composites. Mater. Sci. Eng. A.

[28] Ouyang J.H., Li Y.F., Wang Y.M., Zhou Y., Murakami T., Sasaki S. (2009). Microstructure and Tribological Properties of ZrO_2_(Y_2_O_3_) Matrix Composites Doped with Different Solid Lubricants from Room Temperature to 800 °C. Wear.

[29] Shi X., Yao J., Xu Z., Zhai W., Song S., Wang M., Zhang Q. (2014). Tribological Performance of TiAl Matrix Self-Lubricating Composites Containing Ag, Ti_3_SiC_2_ and BaF_2_/CaF_2_ Tested from Room Temperature to 600 °C. Mater. Des..

[30] Elsheikh A.H., Yu J., Sathyamurthy R., Tawfik M.M., Shanmugan S., Essa F.A. (2020). Improving the Tribological Properties of AISI M50 Steel Using SnS/ZnO Solid Lubricants. J. Alloys Compd..

[31] Zuomin L., Childs T.H.C. (2004). The Study of Wear Characteristics of Sintered High Speed Steels Containing CaF_2_, MnS and TiC Additives at Elevated Temperature. Wear.

[32] Rajkumar K., Aravindan S. (2011). Tribological Performance of Microwave Sintered Copper–TiC–Graphite Hybrid Composites. Tribol. Int..

[33] Cui G., Bi Q., Niu M., Yang J., Liu W. (2013). The Tribological Properties of Bronze–SiC–Graphite Composites under Sea Water Condition. Tribol. Int..

[34] Cui G., Bi Q., Zhu S., Yang J., Liu W. (2012). Tribological Properties of Bronze–Graphite Composites under Sea Water Condition. Tribol. Int..

[35] Rajkumar K., Aravindan S. (2009). Microwave Sintering of Copper–Graphite Composites. J. Mater. Process. Technol..

[36] Cui G., Niu M., Zhu S., Yang J., Bi Q. (2012). Dry-Sliding Tribological Properties of Bronze–Graphite Composites. Tribol. Lett..

[37] Muterlle P.V., Cristofolini I., Pilla M., Pahl W., Molinari A. (2011). Surface Durability and Design Criteria for Graphite–Bronze Sintered Composites in Dry Sliding Applications. Mater. Des..

[38] Ren B., Gao L., Li M., Zhang S., Ran X. (2020). Tribological Properties and Anti-Wear Mechanism of ZnO@Graphene Core-Shell Nanoparticles as Lubricant Additives. Tribol. Int..

[39] Kestursatya M., Kim J.K., Rohatgi P.K. (2003). Wear Performance of Copper–Graphite Composite and a Leaded Copper Alloy. Mater. Sci. Eng. A.

[40] Cui G., Bi Q., Yang J., Liu W. (2013). Fabrication and Study on Tribological Characteristics of Bronze–Alumina–Silver Composite under Sea Water Condition. Mater. Des..

[41] Cui G., Li J., Wu G. (2014). Friction and Wear Behavior of Bronze Matrix Composites for Seal in Antiwear Hydraulic Oil. Tribol. Trans..

[42] Cui G., Liu Y., Gao G., Liu H., Kou Z. (2020). Microstructure and High-Temperature Wear Performance of FeCr Matrix Self-Lubricating Composites from Room Temperature to 800 °C. Materials.

[43] Kato H., Takama M., Iwai Y., Washida K., Sasaki Y. (2003). Wear and Mechanical Properties of Sintered Copper–Tin Composites Containing Graphite or Molybdenum Disulfide. Wear.

[44] Zhang X., Zhang X., Wang A., Huang Z. (2009). Microstructure and Properties of HVOF Sprayed Ni-Based Submicron WS_2_/CaF_2_ Self-Lubricating Composite Coating. Trans. Nonferrous Met. Soc. China.

[45] Zhang X., Zhang L., Huang Z. (2014). Characterization of Ni-based alloy submicron WS_2_/CaF_2_ composite coatings deposited by high velocity oxy-fuel (HVOF) spray process. Adv. Mater. Res..

[46] Yuan J.-H., Zhu Y., Ji H., Zheng X., Ruan Q., Niu Y., Liu Z., Zeng Y. (2010). Microstructures and Tribological Properties of Plasma Sprayed WC–Co–Cu–BaF_2_/CaF_2_ Self-Lubricating Wear Resistant Coatings. Appl. Surf. Sci..

[47] Huang C., Du L., Zhang W. (2013). Friction and Wear Characteristics of Plasma-Sprayed Self-Lubrication Coating with Clad Powder at Elevated Temperatures up to 800 °C. J. Therm. Spray Technol..

[48] Cai B., Tan Y., He L., Tan H., Wang X. (2013). Tribological Behavior and Mechanisms of Graphite/CaF_2_/TiC/Ni-Base Alloy Composite Coatings. Trans. Nonferrous Met. Soc. China.

[49] Yao Q., Jia J., Chen T., Xin H., Shi Y., He N., Feng X., Shi P., Lu C. (2020). High Temperature Tribological Behaviors and Wear Mechanisms of NiAl-MoO_3_/CuO Composite Coatings. Surf. Coat. Technol..

[50] Kobayashi T., Maruyama T., Yasuda T. (2003). Sliding Properties of Composite Sprayed Coating between Bronze Powder and Solid Lubricant. Mater. Trans..

[51] Ling H.J., Mai Y.J., Li S.L., Zhang L.Y., Liu C.S., Jie X.H. (2019). Microstructure and Improved Tribological Performance of Graphite/Copper Zinc Composite Coatings Fabricated by Low Pressure Cold Spraying. Surf. Coat. Technol..

[52] Xiang Z.-F., Liu X.-B., Ren J., Luo J., Shi S.-H., Chen Y., Shi G.-L., Wu S.-H. (2014). Investigation of Laser Cladding High Temperature Anti-Wear Composite Coatings on Ti6Al4V Alloy with the Addition of Self-Lubricant CaF_2_. Appl. Surf. Sci..

[53] Yan H., Zhang J., Zhang P., Yu Z., Li C., Xu P., Lu Y. (2013). Laser Cladding of Co-Based Alloy/TiC/CaF_2_ Self-Lubricating Composite Coatings on Copper for Continuous Casting Mold. Surf. Coat. Technol..

[54] Yan H., Zhang P., Yu Z., Lu Q., Yang S., Li C. (2012). Microstructure and Tribological Properties of Laser-Clad Ni–Cr/TiB_2_ Composite Coatings on Copper with the Addition of CaF_2_. Surf. Coat. Technol..

[55] Liu W.-G., Liu X.-B., Zhang Z.-G., Guo J. (2009). Development and Characterization of Composite Ni–Cr–C–CaF_2_ Laser Cladding on γ-TiAl Intermetallic Alloy. J. Alloys Compd..

[56] Wang H.M., Yu Y.L., Li S.Q. (2002). Microstructure and Tribological Properties of Laser Clad CaF_2_/Al_2_O_3_ Self-Lubrication Wear-Resistant Ceramic Matrix Composite Coatings. Scr. Mater..

[57] Piasecki A., Kulka M., Kotkowiak M. (2016). Wear Resistance Improvement of 100CrMnSi6-4 Bearing Steel by Laser Boriding Using CaF_2_ Self-Lubricating Addition. Tribol. Int..

[58] Piasecki A., Kotkowiak M., Kulka M. (2017). Self-Lubricating Surface Layers Produced Using Laser Alloying of Bearing Steel. Wear.

[59] Piasecki A., Kotkowiak M., Makuch N., Kulka M. (2019). Wear Behavior of Self-Lubricating Boride Layers Produced on Inconel 600-Alloy by Laser Alloying. Wear.

[60] Piasecki A., Kotkowiak M., Tulinski M., Kubiak A. (2022). Tribological Behavior and Wear Mechanism of Ni-Nano TiO_2_ Composite Sintered Material at Room Temperature and 600 °C. Lubricants.

[61] Kong X., Sun W., Wang Q., Chen M., Zhang T., Wang F. (2022). Improving High-Temperature Wear Resistance of NiCr Matrix Self-Lubricating Composites by Controlling Oxidation and Surface Texturing. J. Mater. Sci. Technol..

[62] Houdková Š., Smazalová E., Vostřák M., Schubert J. (2014). Properties of NiCrBSi Coating, as Sprayed and Remelted by Different Technologies. Surf. Coat. Technol..

[63] Appiah A.N.S., Bialas O., Żuk M., Czupryński A., Sasu D.K., Adamiak M. (2022). Hardfacing of Mild Steel with Wear-Resistant Ni-Based Powders Containing Tungsten Carbide Particles Using Powder Plasma Transferred Arc Welding Technology. Mater. Sci. Pol..

[64] Praveen A.S., Arjunan A. (2022). High-Temperature Oxidation and Erosion of HVOF Sprayed NiCrSiB/Al_2_O_3_ and NiCrSiB/WC Co Coatings. Appl. Surf. Sci. Adv..

